# Extended-duration thromboprophylaxis after ventral hernia repair: a risk model to predict venous thrombotic events after hospital discharge

**DOI:** 10.1007/s10029-021-02481-x

**Published:** 2021-08-15

**Authors:** S. B. Kumar, D. Mettupalli, J. T. Carter

**Affiliations:** 1 Department of Surgery, University of California San Francisco-Zuckerberg San Francisco General Hospital, 1001 Potrero Avenue, Suite 3A, San Francisco, CA 94110 USA; 2 University of California, Berkeley, Berkeley, CA USA; 3 Department of Surgery, University of California San Francisco, San Francisco, CA USA

**Keywords:** Ventral hernia repair, Venous thromboembolism, Risk prediction, Thromboprophylaxis

## Abstract

**Background:**

Venous thromboembolism (VTE) is a major source of morbidity and mortality after ventral hernia surgery, but the risk of VTE after discharge has not been reported.

**Study design:**

Data from the American College of Surgeons-National Surgical Quality Improvement Program (ACS-NSQIP) were used to investigate the risk of post-discharge VTE. Current procedural terminology (CPT) codes identified all reported patients who underwent ventral hernia repair from 2011 to 2017. We created a multivariable regression model for post-discharge VTE, using the 2011–2016 dataset to develop the model and 2017 as a validation set. The prediction model was used to create a risk calculator as a mobile application.

**Results:**

The rate of VTE after surgery was 0.62% (878 of 141,065) with 48% occurring after discharge from the hospital. The final predictor model consisted of eight variables: age > 60 years, male sex, body mass index (BMI) ≥ 35 kg/m^2^), operative time > 2 h, concurrent panniculectomy, post-operative hospitalization > 1 day, presence of bleeding disorder, and emergency operation. The model had good calibration and discrimination (Hosmer–Lemeshow goodness-of-fit test, *p* = 0.71; *c*-statistic = 0.71). Threshold analysis showed a strategy of extended-duration thromboprophylaxis was optimized when the risk of post-discharge VTE was > 0.3%.

**Conclusion:**

Forty-eight percent of VTEs after ventral hernia repair occur after discharge, particularly in older, male, obese patients undergoing longer and complex operations that require hospitalization > 1 day. Post-discharge thromboprophylaxis should be considered in these patients, particularly when risk of VTE exceeds 0.3%.

## Introduction

Venous thromboembolism (VTE), defined as a deep vein thrombosis, pulmonary embolus, or both, remains a major source of morbidity and mortality after abdominal surgery [1]. Hernia patients are no exception [2, 3]. Because one-third of all VTEs occur after surgery and effective risk-reduction strategies exist, thromboprophylaxis strategies should be considered after every surgical procedure. Current CHEST guidelines recommend either mechanical thromboprophylaxis (such as sequential compression devices), chemothromboprophylaxis (i.e., unfractionated heparin, low-molecular weight heparin, or other agents); or both—depending on the degree of thrombotic risk and the overall risk of bleeding. For most patients, including hernia patients, CHEST guidelines recommend these prophylactic strategies to continue only while the patient is hospitalized, under the assumption that once a patient can walk easily after discharge, their risk of VTE has returned to baseline [1].

In the past decade, however, this assumption has been challenged. Many VTEs following surgery occur after discharge from the hospital in patients who are presumably walking [4–7]. Some reports have shown increased risk of VTE up to 90 days after surgery [3, 5]. For example, in patients undergoing bariatric surgery, one report found that 80% of VTEs occurred after discharge [5]. Cancer patients undergoing abdominopelvic operations are known to be at high risk for post-discharge VTE [8]. As a result, current guidelines for abdominopelvic cancer patients call for extended-duration thromboprophylaxis for 4 weeks after surgery, irrespective of the date of discharge from the hospital or ambulatory status [1, 8].

Ventral hernia patients are known to be at risk for VTEs [2, 3] but there have been no reports examining the risk of VTE after discharge from the hospital, and whether risk factors can be identified to select hernia patients who may benefit from extended-duration thromboprophylaxis. In this study, we used data from the American College of Surgeons-National Surgical Quality Improvement Program (ACS-NSQIP) to investigate the risk of post-discharge VTE. We then identified which patients may be suitable for extended-duration thromboprophylaxis by creating a risk calculator, and validating the calculator on a separate cohort of patients.

## Methods

### Patient selection

ACS-NSQIP prospectively collects procedural information from about 700 hospitals in the United States and Canada and includes patient characteristics, operative variables, complications, and mortality within 30 days of a surgical procedure. We identified all patients who underwent ventral hernia repair reported to ACS-NSQIP from 2011 to 2017 using current procedural terminology (CPT) codes. CPT codes are used in the United States to report medical, surgical, and diagnostic procedures and services to entities such as physicians, health insurance companies, and accreditation organizations. We used following codes: open ventral hernia repair: 49560, 49561, 49565, 49566; laparoscopic ventral hernia repair: 49654, 49655, 49656, 49657; and implantation of mesh: 49568, 15777, 15330, 15331, 15430, 15431. Concurrent procedures were also identified by CPT codes: panniculectomy: 15830; myocutaneous flap: 15734, 15738; concurrent abdominal procedure: 49000, 44005, 47600, 47605, 47563, 44121, 44125, 44130, 44120, 44202, 44602, 44603, 44020, 44227, 44640, 44620, 44346, 58150, 58940; and concurrent bowel procedure: 44121, 44125, 44130, 44120, 44202, 44602, 44603, 44020, 44227, 44640, 44620, 44346. Hernia characteristics were also identified: recurrent hernia: 49565, 49566, 49656, 49657; and incarcerated/strangulated: 49561, 49566, 49655, 49657. The primary outcome was development of a post-discharge VTE, defined as either a deep vein thrombosis or pulmonary embolus that occurred after discharge from the hospital, but within the NSQIP 30-day follow-up period. Deep vein thrombosis was defined in NSQIP as a venous thrombosis, confirmed by a duplex, venogram, CT scan, or other definitive imaging modality, or autopsy; treated with anticoagulation therapy, vena cava filter, clipping of the vena cava, or documentation that treatment was warranted but there was no appropriate treatment option available, or that the patient refused treatment. Pulmonary emboli were defined in NSQIP as new blood clot in a pulmonary artery causing obstruction of the blood supply to the lungs and diagnosed with a V-Q scan interpreted as high probability of pulmonary embolism, trans-esophageal echocardiogram, pulmonary arteriogram, CT angiogram, or any other definitive imaging modality (including autopsy). As this study was performed on a nationally available deidentified dataset, it was exempt from Institutional Review Board Approval.

### Model development

We then created a model that included patient characteristics and procedural details to predict post-discharge VTEs. Patient characteristics included age, Body Mass Index (BMI), sex, race, diabetes mellitus, smoking history, dyspnea, functional status, ventilator dependence, chronic obstructive pulmonary disease, ascites, congestive heart failure, hypertension, acute renal failure, dialysis dependence, disseminated cancer, pre-existing open wound, chronic steroid use, bleeding disorder, preoperative transfusion, preoperative sepsis, total hospital length of stay, days from operation to discharge, discharge destination, and American Society of Anesthesiologists class. Procedural details were operative time, laparoscopic versus open approach, recurrent hernia, incarcerated or strangulated hernia, mesh use, need for myocutaneous flap, panniculectomy, concurrent abdominal procedure, and concurrent bowel procedure. Preoperative laboratory values, such as serum creatinine, albumin, hematocrit, and platelet count, were not included as possible predictors, since there were large amounts of missing data. The NSQIP data set does not contain information as to the thromboprophylaxis strategies (i.e., mechanical versus chemothromboprophylaxis) employed during hospitalization or after discharge. We also assessed reoperation, readmission, and death within 30 days.

### Statistical analysis

Baseline characteristics and outcomes were compared using the Wilcoxon-rank sum test for continuous variables and Pearson *χ*^2^ for categorical variables. The risk model was constructed using a primary data set of patients who underwent ventral hernia repair from 2011 to 2016 (*n* = 140,301) and validated using the cohort of patients undergoing procedures in 2017 (*n* = 29,816). Predictors with *p* value < 0.05 in univariate analysis, as well as those known to be associated with VTE were used to construct a multivariable model using forward and backward stepwise regression. The calibration of the model was tested using the Hosmer–Lemeshow goodness-of-fit test, and the discriminatory ability was evaluated using the *c*-statistic. Various cut-points for the calculated risk of post-discharge VTE were assessed using ROC curves, sensitivity, specificity, and Youden’s *J* index (defined as sensitivity + specificity − 1). An equation to calculate the predicted risk of post-discharge VTE was then created from the model and incorporated into a mobile application. All data analysis was performed using STATA/MP 16.0 (StataCorp, College Station, TX).

## Results

### Risk of post-operative VTE

**Fig. 1:**
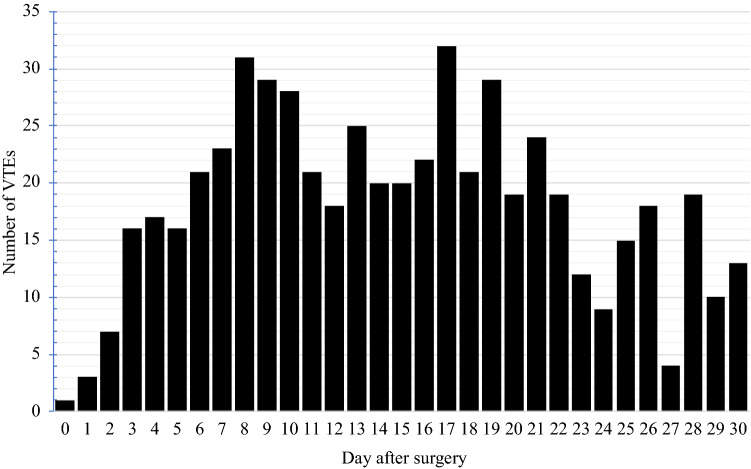
Histogram of the number of VTEs that occurred on each post-operative day after ventral hernia repair, based on data from the American College of Surgeons-National Surgical Quality Improvement Program (ACS-NSQIP) for the years 2011–2017

VTEs occurred in 878 of 141,065 patients (0.62%) within 30 days of surgery. About half of these were deep venous thromboses (0.33%) and the other half pulmonary emboli (0.35%). When we examined the timing of VTEs relative to hospital discharge, about half (421, or 48%) of VTEs occurred after discharge from the hospital. Again, the distribution of these post-discharge VTEs was evenly split between deep vein thrombosis (245, 58%) and pulmonary embolus (176, 42%). We then examined the timing of all VTEs relative to the primary hernia operation (Fig. 1). The risk of VTE did not appreciably begin to decrease until 21 days after surgery and was still present at 30 days.

### Comparison of patients with post-discharge VTE to those without

Baseline characteristics of the study cohort, excluding 764 patients who suffered a VTE during their index hospitalization or were still hospitalized at 30 days, are summarized in Table 1. Patients who developed post-discharge VTE were older (61 vs. 57 years, *p* < 0.001), more obese, (BMI 34 vs. 32 kg/m^2^, *p* < 0.001), were more likely to be discharged someplace other than home, and had higher rates of comorbidities: dyspnea, impaired functional status, COPD, CHF, hypertension, cancer, open wounds, bleeding disorders, higher American Society of Anesthesiology class, and more preoperative systemic inflammatory response syndrome. Patients who suffered a post-discharge VTE also had a much longer length of stay in the hospital after their hernia operation (4 vs. 1 days, *p* < 0.001).

We then compared operative details between cohorts (Table 2). Patients with post-discharge VTE had significantly longer operations (2.1 vs. 1.3 h, *p* < 0.001), and were more likely to be inpatient (73% versus 46%, *p* < 0.001), emergent (23% versus 12%, *p* < 0.001), and to have a higher wound class (*p* < 0.001). A laparoscopic approach protected patients from post-discharge VTE (15% versus 21%, *p* = 0.007). Hernia characteristics that increased operative complexity increased the rate of post-discharge VTE: incarceration or strangulation (41% versus 33%, *p* < 0.001), need for mesh (52% versus 46%, *p* = 0.011), myocutaneous flap (14% versus 7%, *p* < 0.001), panniculectomy (4% versus 1%, *p* < 0.001), or concurrent abdominal (22% versus 12%, *p* < 0.001) or bowel procedure (5% versus 2%, *p* < 0.001). Finally, patients who experienced a post-discharge VTE had a higher rate of reoperation (14% versus 2%, *p* < 0.001), readmission (73% versus 6%, *p* < 0.001), and 30-day mortality (1.4% versus 0.4%, *p* < 0.001).

**Table 1:** Characteristics of patients with ventral hernia repair who developed a VTE after discharge

Patient characteristic	No VTE (*n* = 139,880)	Post-discharge VTE (*n* = 421)	*p* value
Mean age (years)	57 (47–67)	61 (52–70)	< 0.001
Body mass index (kg/m^2^)	31.6 (27.4–37.0)	34.1 (29.4–40.1)	< 0.001
Female sex (%)	80,280 (57.4)	222 (52.7)	0.15
Discharge destination (%)			< 0.001
Home	134,803 (96.6)	387 (92.1)	
Facility which was home	732 (0.5)	1 (0.2)	
Skilled or unskilled facility	2498 (1.8)	26 (6.2)	
Rehabilitation	855 (0.6)	4 (1.0)	
Separate acute care	349 (0.3)	2 (0.5)	
Hospice	3 (0.0)	0 (0.0)	
Other	357 (0.3)	0 (0.0)	
Diabetes (%)			0.48
No diabetes	115,078 (82.3)	337 (80.1)	
Not on insulin	16,543 (11.8)	57 (13.5)	
Taking insulin	8259 (5.9)	27 (6.4)	
Smoker (%)	26,862 (19.2)	70 (16.6)	0.18
Dyspnea (%)	9.334 (6.8)	45 (10.7)	0.001
Impaired functional status (%)	1860 (1.3)	14 (3.3)	< 0.001
Ventilator dependent (%)	97 (0.1)	2 (0.5)	0.002
Chronic Obstructive Pulmonary Disease (%)	7754 (5.5)	37 (8.8)	0.004
Ascites (%)	711 (0.5)	4 (1.0)	0.204
Congestive heart failure (%)	817 (0.6)	4 (1.0)	0.325
Hypertension (%)	70,268 (50.2)	244 (58.0)	0.002
Acute renal failure (%)	293 (0.2)	1 (0.2)	0.9
Dialysis dependence (%)	1462 (1.1)	3 (0.7)	0.5
Cancer (%)	1007 (0.7)	5 (1.2)	0.257
Open wound (%)	1995 (1.4)	16 (3.8)	< 0.001
On chronic steroids (%)	5993 (4.3)	20 (4.8)	0.64
Bleeding disorder (%)	4446 (3.2)	31 (7.4)	< 0.001
Preoperative transfusion (%)	236 (0.2)	0 (0.0)	0.4
Systemic Inflammatory Response Syndrome (%)	3289 (2.4)	21 (5.0)	< 0.001
Hospital days after surgery (range)	1 (0–3)	4 (1–7)	< 0.001
American Society of Anesthesiology Class			< 0.001
Class I (%)	7750 (6)	7 (2)	
Class II (%)	63,635 (46)	137 (33)	
Class III (%)	63,329 (45)	254 (60)	
Class IV (%)	4971 (4)	23 (6)	
Class V (%)	33 (0.02)	0 (0)	

### Construction of predictor model

Stepwise multivariate regression of predictors resulted in the final multivariable model of eight independent risk factors for prediction of post-discharge VTE (Table 3). Calibration of the model was measured by the Hosmer–Lemeshow goodness-of-fit test, *χ*^2^ = 360.19 (*p* = 0.711), and the discrimination of the model in the training data set was measured by the *c*-statistic = 0.71. The model was then validated in the 2017 dataset, consisting of 29,816 patients, and the *c*-statistic was 0.64 (95% CI 0.58–0.69). The equation generated by the multivariable model takes the form of $\hat{p}$ = exp (*b*_0_ + *b*_1_ + *b*_2_ + *…* + *b*_*p*_)/[1 + exp (*b*_0_ + *b*_1_ + *b*_2_ + *…* + *b*_*p*_)], where $\hat{p}$ is the predicted risk of post-discharge VTE, *b*_*0*_ is the constant (− 7.12), and *b*_1_, *b*_2_, … *b*_p_ are the coefficients for the logit function. This regression equation is the basis for a risk calculator in the form of a free and easy to use mobile application for both iOS (https://apps.apple.com/us/app/ventral-hernia-vte-risk/id1557362906) and Android devices (https://play.google.com/store/apps/details?id=com.ucsf.crpapp).

**Table 2:** Operative details of patients with ventral hernia repair who developed VTE after discharge from the hospital

Operative characteristic	No VTE (*n* = 139,880)	Post-discharge VTE (*n* = 421)	*p* value
Mean operative time in hours (range)	1.3 (0.7–2.1)	2.1 (1.2–3.3)	< 0.001
Admission required			< 0.001
Outpatient (%)	75,206 (54)	113 (27)	
Inpatient (%)	64,674 (46)	308 (73)	
Emergency operation (%)	17,339 (12)	97 (23)	< 0.001
Wound class			< 0.001
Clean (%)	122,660 (88)	333 (79)	
Clean contaminated (%)	12,765 (9)	61 (15)	
Contaminated (%)	2431 (2)	18 (4)	
Dirty/Infected (%)	2024 (2)	9 (2)	
Laparoscopic approach (%)	28,772 (21)	64 (15)	0.007
Recurrent hernia (%)	30,661 (22)	107 (25)	0.083
Incarcerated or strangulated hernia (%)	45,483 (33)	173 (41)	< 0.001
Mesh used (%)	64,163 (46)	219 (52)	0.011
Myocutaneous flap (%)	9028 (7)	59 (14)	< 0.001
Panniculectomy (%)	1649 (1)	16 (4)	< 0.001
Concurrent abdominal procedure (%)	16,567 (12)	92 (22)	< 0.001
Concurrent bowel procedure (%)	2636 (2)	19 (5)	< 0.001
Reoperation (%)	3347 (2)	60 (14)	< 0.001
Readmission (%)	8053 (6)	306 (73)	< 0.001
Death within 30 days (%)	528 (0.4)	6 (1)	< 0.001

### Selection of risk threshold to consider extended-duration thromboprophylaxis

The predictors listed in Table 3 allow for the calculation of a given patient’s risk of post-discharge VTE. For instance, the formula might estimate a given patient’s risk of post-discharge VTE to be 0.65% based on the presence or absence of predictors. The next question becomes, at what risk threshold should surgeons consider extended-duration thromboprophylaxis? To investigate, we evaluated the performance of the risk prediction model in our validation data set using different thresholds (Table 4). For each threshold, we calculated the sensitivity, specificity, Youden’s J index, and the AUC. For example, a threshold risk of > 0.3% would include 36% of all hernia patients, have a sensitivity of 55%, and have a specificity of 64% for predicting post-discharge VTE. If a higher threshold of > 0.4% was chosen, only 25% of all hernia patients would be included, the sensitivity would decrease to 34%, while the specificity would increase to 75%.

**Table 3:** Multivariable analysis of factors predictive of post-discharge VTE

Risk Factor	Odds ratio (95% CI)	*p* value	Logit coefficient
Age > 60 years	1.43 (1.17–1.74)	< 0.0001	0.36
Male sex	1.42 (1.16–1.73)	0.001	0.35
Body mass index (referenced to BMI < 35 kg/m^2^)			
35–40 kg/m^2^	1.36 (1.06–1.76)	0.017	0.31
40–45 kg/m^2^	1.57 (1.14–2.15)	0.005	0.45
45–50 kg/m^2^	1.86 (1.25–2.77)	0.002	0.62
> 50 kg/m^2^	1.64 (1.12–2.43)	0.013	0.50
Operative time > 2 h	1.92 (1.54–2.38)	< 0.0001	0.65
Panniculectomy performed	2.01 (1.20–3.37)	0.008	0.7
Postoperative hospitalization > 1 day	2.27 (1.76–2.91)	< 0.0001	0.82
Bleeding disorder^a^ present	1.68 (1.15–2.44)	0.007	0.52
Emergency operation	1.43 (1.11–1.83)	0.005	0.35

^a^ACS-NSQIP defines bleeding disorder as any chronic condition that places the patient at risk for excessive bleeding (e.g., vitamin K deficiency, hemophilia, thrombocytopenia, chronic anticoagulation therapy that has not been discontinued prior to surgery, heparin-induced thrombocytopenia, and so on, excluding aspirin therapy and nonsteroidal anti-inflammatory drug use). To calculate the risk of post-discharge VTE, add up the logit function coefficients for each factor present, plus an additional constant of − 7.12. The sum predicts the probability of post-discharge VTE by the logit function, equal to *e*^sum^/(1 + *e*^sum^). This calculation is facilitated by a mobile application

**Table 4:** Sensitivity, specificity, and Youden’s *J* performance for different thresholds of post-discharge VTE risk

Threshold (%)	# Patients	Sensitivity (95% CI)	Specificity (95% CI)	Youden’s *J*	AUC
≥ 0.1	26,914 (90%)	96% (89–99)	10% (9–10)	0.055	0.53 (0.51–0.55)
≥ 0.2	14,274 (48%)	71% (61–80)	52% (52–53)	0.23	0.62 (0.57–0.66)
≥ 0.3	10,657 (36%)	55% (44–65)	64% (64–65)	0.19	0.6 (0.54–0.65)
≥ 0.4	7536 (25%)	34% (25–45)	75% (74–75)	0.09	0.55 (0.5–0.59)
≥ 0.5	5904 (20%)	32% (23–43)	80% (80–81)	0.13	0.56 (0.51–0.61)
≥ 0.75	2232 (7%)	17% (10–26)	93% (92–93)	0.10	0.55 (0.51–0.59)
≥ 1	982 (3%)	9% (4–16)	97% (96–97)	0.05	0.53 (0.50–0.56)
≥ 3	35 (0.1%)	1% (0.0–6)	99% (99–100)	0.01	0.5 (0.49–0.52)

## Discussion

In this study, we found that one VTE occurred for roughly every 160 patients (0.62%) who underwent ventral hernia repair. VTEs conferred serious morbidity; they were associated with a readmission in the majority of cases and 2.5-fold increased odds of death. Importantly, almost half of the VTEs (48%) occurred after hospital discharge. Based upon this observation, it seems premature to discontinue thromboprophylaxis in high-risk patients at the time of discharge from the hospital—an arbitrary point in time with respect to VTEs. Indeed, our analysis found that the overall risk of VTE did not begin to diminish until 21 days after the operation and persisted out to 30 days (Fig. 1).

This is not to say that all hernia patients are at high risk for post-discharge VTEs and need extended-duration thromboprophylaxis. We identified eight independent predictors of post-discharge VTE based upon the patient’s age, gender, obesity, complexity of hernia repair, and length of stay (Table 3). These predictors align with clinical experience. For example, a 75-year-old man with a BMI 41 kg/m^2^, undergoing a 4-h open lysis of adhesions and abdominal wall reconstruction with panniculectomy and requiring a 7-day hospital stay would have a much greater risk of post-discharge VTE (2.2%) compared to a 45-year-old woman with normal BMI undergoing outpatient ventral hernia repair with operative time < 2 h (0.08%). Notably, technical factors such as longer operative time and need for panniculectomy are also in line with the predictor model for all VTE published by Pannucci [2]. We did not find any association between myocutaneous flap component separation and post-discharge VTE, similar to other studies [9]. Although the final *c*-statistic of the predictive model was 0.64, meaning that some post-discharge VTEs could not be predicted from the model, the overall accuracy was in line with other predictive models currently in use [2, 5]. Additionally, the simplicity of the model adds to its clinical usefulness.

We developed and published a mobile application that surgeons can download into their smartphone (freely downloadable on iOS App Store or Android: “Ventral Hernia VTE Risk Calculator”). The application calculates the post-discharge VTE risk for a given patient. It is our hope that surgeons will use the application to risk-stratify patients and decide whether extended-duration thromboprophylaxis may be of benefit. This approach has proved successful for bariatric surgeons who use the BariatricCalc application, published by the Cleveland Clinic [5].

At what threshold of risk should surgeons consider extended-duration thromboprophylaxis in their ventral hernia patients? This decision should be based upon four factors: (1) the absolute risk of post-discharge VTE without thromboprophylaxis, (2) the number of patients needed to treat to avoid one post-discharge VTE, (3) the risk of adverse events, particularly bleeding, from prescribing extended-duration thromboprophylaxis, and (4) the cost of therapy. Our study addresses the first two factors. To estimate the third factor, the risk of bleeding with thromboprophylaxis, a recent Cochrane analysis estimated the odds of post-operative hemorrhage associated with extended-duration thromboprophylaxis to be 1.1 (95% confidence interval 0.67–1.81). This very low odds of bleeding was not statistically significant, meaning that extended-duration thromboprophylaxis was not associated with additional bleeding events [10]. As such, the risk of adverse events specifically from extended-duration thromboprophylaxis seems minimal. For the fourth factor, cost of therapy, most centers utilize low-molecular weight heparin in their extended-duration thromboprophylaxis protocols. Unfortunately, these agents are expensive, and often not covered by insurance. As a result, prophylaxis of a large percentage of ventral hernia patients may not be practical [11, 12].

To balance factors, we recommend surgeons consider prescribing extended-duration thromboprophylaxis when the calculated risk of post-discharge VTE exceeds 0.3%. This approach would require prophylaxis for only a third of ventral hernia patients (36%), but this cohort would include over half (55% sensitivity, 64% specificity) of the patients who would otherwise develop post-discharge VTEs. At this threshold, the number needed to treat to prevent one post-discharge VTE would be 200. If the cost of therapy were to decrease and no longer be a factor in decision-making, a threshold of 0.2% risk would include almost half (48%) of ventral hernia patients to get prophylaxis, but this cohort would include almost three quarters (71% sensitivity, 52% specificity) of the patients destined to develop post-discharge VTE. Statistically, the Youden’s *J*-statistic also calculates the optimal threshold, based upon the characteristics of the predictor model alone (not taking into account cost, or risk of adverse events). In our study, Youden’s *J*-statistic was optimized at a threshold risk of 0.2–0.3% (*J*-statistics 0.23, 0.19, Table 4), in line with our recommended threshold of 0.3%.

To see if a specific patient’s post-discharge VTE risk exceeded 0.3%, surgeons would use the mobile application and enter whether any of the 8 predictors were present. For example, the first patient described above, would have a calculated risk of post-discharge VTE of 2.2%, and would therefore be suitable for extended-duration thromboprophylaxis after discharge. We propose 4 weeks of thromboprophylaxis based upon the observation that risk does not appreciably decrease until post-operative day 21 (Fig. 1). This is in line with recommendations for other high-risk patients undergoing extended-duration thromboprophylaxis [4–6, 8].

Our study has several limitations. First, ACS-NSQIP only tracks outcomes within 30 days of surgery. VTE events that occurred later were not captured in the data set, and as such, the true cumulative VTE rate is probably higher than reported here. Second, ACS-NSQIP does not capture all hernia-specific factors that could be predictive of post-discharge VTE, such as hernia dimensions or loss of domain; operative time, need for myocutaneous flap, and panniculectomy serve as surrogates for complexity and size. Third, construction of a predictor model would ideally test all of the variables used in the Caprini scoring system, which is the most popular VTE risk assessment tool today [1, 13–15]. Unfortunately, many of the Caprini risk factors are not contained in ACS-NSQIP, and therefore not available for analysis. Finally, it is impossible to identify which patients received thromboprophylaxis, and what type of prophylaxis they received, both after surgery and after discharge. This information is not contained in ACS-NSQIP. We presume that many patients received prophylaxis while hospitalized, but the number that may have received after discharge is unknown. Hence, our estimate of VTE risk in hospitalized patients is probably less than the true risk would be without any thromboprophylaxis. Nevertheless, despite these limitations, the strength of this study lies in the clinical simplicity of the scoring system and validation using a separate test cohort containing a very large number of hernia patients.

## Conclusions

Almost half of venous thromboembolic events after ventral hernia repair occurred after discharge from the hospital, and the risk of an event did not start to decline until three weeks after surgery. Patients at high risk for post-discharge VTE can be identified based upon patient and procedural characteristics using a simple clinical predictor tool and mobile application. When the risk exceeds 0.3%, extended-duration thromboprophylaxis for 4 weeks can be considered as a strategy to reduce VTE events.
